# Botanical Description of British Plants

**Published:** 1807-06

**Authors:** 


### *Botanical Description of British Plants.*

[ Continued from our last, page 464—469. ]

7. MENTHA. *M. viridis.*

*Ang.* Spear-mint. Mint.

*Gen. Desc.* Bloss. nearly equal; four-cleft; the broader segments notched at the end. Stamens upright, distant.

*Spec. Desc.* *Flowers* in spikes. *Sp.* oblong. *Leaves* spear-shaped, naked, serrated, sitting. *Bloss.* purplish red. *Watery places, banks of rivers.* BL. July, Aug.

*Use.* To spear-mint are to be ascribed the same medicinal qualities which belong to pepper-mint; but the preparations of it, though pleasanter, are less efficacious than those of the latter. It contains much essential oil, but of an odour somewhat less agreeable than that of lavender or marjoram. It is therefore less employed as a *cephalic;* but it acts very powerfully on the parts to which it is immediately applied, and therefore considerably on the stomach, invigorating all its functions. It acts especially as an *antispasmodic,* and therefore relieves pains and cholic depending depending upon spasm: it will also stop vomiting depending upon such a cause; but there are many cases of vomiting in which it is of no service; and in these cases, anywise depending upon inflammatory irritation in the stomach itself, or in other parts of the body, it aggravates the disease, and increases the vomiting. Practitioners have thought, and justly according to the opinion of Dr. Woodville, that the infusion of mint in warm water, agrees better with the stomach than the distilled water, which is often somewhat empyreumatic.—*Cullen ii.* 149.—Mint, according to Linnæus, possesses so strong an *emmenagogue* power, that a woman, by the frequent use of it, became subject to menorrhagia. *Diss. de M. usu, p.* 9. Lewis observes, "that it is said by some, to prevent the coagulation of milk, and hence it has been recommended to be used along with milk diets, and even in cataplasms and fomentations for resolving coagulated milk in the breasts: upon experiment, the curd of milk, digested in a strong infusion of mint, could not be perceived to be any otherwise affected than by common water; but milk, in which mint leaves were set to macerate, did not coagulate near so soon as an equal quantity of the same milk kept by itself." *M. M. p.* 419. The officinal preparations are an essential oil, a conserve, a simple water, and a spirit.—*Woodville.* The flavor of this species being more agreeable than that of the others, it is generally preferred for culinary and medicinal purposes. A conserve of the leaves is very grateful; and the distilled waters, both simple and spirituous, are universally thought pleasant. The leaves are used in spring sallads; and the juice of them, boiled up with sugar, is formed into tablets. The distilled waters and the essential oil, are often given to stop retchings, and frequently with success. — Dr. Lewis says, "that dry mint, digested in rectified spirits of wine, gives out a tincture, which appears by day-light of a fine dark green, but by candle-light of a bright red colour:"— the fact is, that a small quantity of this tincture is green either by day-light or by candle-light, but a large quantity of it seems impervious to common day-light; however, when held between the eye and a candle, or between the eye and the sun, it appears red, so that if put into a flat bottle it appears either green or red, as it is viewed through the flat side, or through the edge of the bottle.—*Withering.*

8. MENTHA. *M. piperita.*

*Ang.* Peppermint.

*Gen. Desc.* *As above.*

*Spec. Desc.* *Leaves* egg-shaped, on leafstalks ; *Stamens* shorter than the blossom. *Flowers* in terminating spikes, separated in clusters, sometimes in whirls, small, reddish purple. Under each whirl two pointed, spear-shaped, hairy floral leaves. *Var.* Leaves spear-shaped. *Watery places.* Bl. Aug. Sept.

*Use.* The spontaneous growth of this plant is said to be peculiar to Britain, but, for medicinal uses, its cultivation has long been extended over Europe. It has a more penetrating smell than any of the other mints, a much stronger and warmer taste, pungent and glowing like pepper, sinking as it were into the tongue, and followed by a sensation of coldness. By maceration or infusion, it readily and strongly impregnates both water and spirit with its virtue. In distillation with water, it yields a considerable quantity of essential oil, of a pale greenish yellow colour, growing darker coloured by age, very light, subtle, possessing in a high degree, the specific smell and penetrating pungency of peppermint.—*Lewis.* Rectification, Dr. Cullen observes, is particularly necessary and proper for this essential oil; What has been called Essence of Peppermint, seems to be no other than the rectified oil dissolved in spirit of wine. *Cullen M. M. ii.* 150. Its *stomachic, antispasmodic,* and *carminative* qualities, render this plant useful in flatulent *cholics, hysterical* affections, *retchings,* and other *dyspeptic* symptoms, acting as a cordial, and often producing immediate relief. Camphor is to be obtained from this plant.—*Woodville.* Peppermint water is well known as a carminative and antispasmodic. The stem and leaves of the plant are beset with numbers of very minute glands, containing the essential oil, which rises plentifully in distillation. The Essence of Peppermint is an elegant medicine, and possesses the most active properties of the plant.—*Withering.*

9. Mentha. *M. arvensis.—M. Sativa. var. M. rubra.*
*Ang.* Cornmint.
*Gen. Desc.* *As above.*

*Spec. Desc.* *Leaves* egg-shaped, acute, serrated, towards the bottom roundish. *Stem* spreading, not tinged with red ; *whole pl.* hairy. *Flowers* in whirls, lateral, *wh.* two opposite umbels, sitting. *Bloss.* hairy within and without, lower segment blunt. *Cal.* pale green, hoary, interspersed with very minute semi-transparent glands. *In watery places and moist corn fields.* Bl. July, Sept.

*Use.* This plant prevents the coagulation of milk; and when cows have eaten it, as they will do largely at the end of of summer, when pastures are bare and hunger distresses them, their milk can hardly be made to yield cheese; a circumstance which sometimes puzzles the dairy-maids.—*Withering.*

10. MENTHA. *M. Pulegium.—Pulegium latifolium.—P. regium.*

*Ang.* Pennyroyal mint.

*Gen. Desc.* *As above.*

*Spec. Desc.* *Leaves* egg-shaped, blunt, somewhat scolloped, thick, slightly toothed, underneath set with deep semi-transparent dots. *Stems* roundish, creeping, four blunt corners, hairy, branched. *Bloss.* twice as long as the calyx, hairy without, pale purple. *Moist heaths and pastures.* BL. Aug. Sept.

*Use.* It has a warm pungent flavor, somewhat similar to mint, but more acrid, and less agreeable both in smell and taste: its active principle is an essential oil of a more volatile nature than that of mint. Pennyroyal possesses the general properties of the other mints; but it is supposed to have less efficacy as a stomachic, though more useful as a *carminative* and *emmenagogue,* and more commonly employed in *hysterical* affections. We are told by Boyle, *v. iv. p.* 475, and others, that it has been successfully used in the *hooping-cough*; but the chief purpose to which it has long been applied, is promoting the uterine evacuation. With this intention, Haller recommends an infusion of the herb with steel in white wine, which, he adds, "*me nunquam fefellit.*" Dr. Cullen, however, considers mint in every respect a more effectual remedy than Pennyroyal, *M. M. ii.* 150; and it is now less frequently used than formerly.—*Woodville.* The expressed juice with a little sugar, is not a bad medicine in the *hooping-cough.* A simple and a spirituous water, distilled from the dried leaves, are kept in the shops: they are prescribed in hysterical affections, and are not without considerable antispasmodic properties: an infusion of the plant may be used with the same intention.—*Withering.*

11. GLECOMA. *G. hederacea.—Hedera terrestris.—Chamæcissus.*

*Ang.* Ground-ivy. Gill. Cat's-foot. Ale-hoof. Tun-hoof. Robin run in the hedge.

*Gen. Desc.* Cal. five-cleft. Anthers in pairs; each pair forming a cross.

*Spec. Desc.* *Leaves* kidney, or heart-shaped, scolloped, underneath hollow dots, in which are glands secreting an essential essential oil, and above, little eminences not secreting any odoriferous oil. *Stamens* sometimes imperfect. *Roots* sending out trailing suckers. *Bloss.* blue, rarely flesh colour. *Groves, shady places.* Bl. April, May.

*Use.* It has a peculiar strong smell; and a bitterish taste, somewhat aromatic. It was formerly supposed to possess great medicinal powers, which later experience has been unable to discover: it is now omitted in the London Mat. Med. Its qualities have been described as *pectoral, detergent, aperient, diuretic, vulnerary, corroborant, errhine, &c.* and has been variously recommended for the cure of diseases to which these powers seem adapted, but chiefly in *pulmonary* and *nephritic* complaints. In obstinate coughs, it is a favourite remedy with the poor, who probably experience its good effects, since they still persevere in its use. Ray, Mead, and others, speak of its being useful joined with fermenting ale; but Dr. Cullen observes that it 'appears to him frivolous: and says, he never has had any evidence of its diuretic or of its pectoral effects: in common with many other of the verticillatæ, he thinks it may be employed as an *errhine,* and in that way cure a head-ache, but no other ways by any specific quality.' Ray gives a remarkable instance of its efficacy in this way, in the complete cure of a most violent cephalalgia. It is usually taken in the way of infusion, or drank as tea. *Woodville.* The leaves thrown into a vat with ale, clarify it, and give it a flavor: the ale thus prepared, is often drank as an *antiscorbutic.*—The expressed juice mixed with a little wine, and applied morning and evening, destroys the white specks upon horses eyes.—Plants that grow near it, do not flourish. It is said to be hurtful to horses, if they eat much of it, but they are not fond of it; sheep eat it; cows, goats, and swine refuse it.—*Withering.* Tea made of the leaves, and sweetened with honey or sugar, is a good remedy for a cough.—*H. B.*

12. Betonica. *B. officinalis. B. purpurea.*

*Ang.* Wood Betony.

*Gen. Desc.* Calyx awned. Bloss. upper lip upright, flat. tube cylindrical.

*Spec. Desc. Spike* interrupted. *Bloss.* purple, upper lip entire, lower lip middle segment notched. Calyxes smoothish. *Stem* square, hairy. *Root leaves* oblong-heart-shaped, scolloped, hairy. *Stem-l.* distant, spear-shaped, serrated. *Woods, shady groves.* Bl. July, Aug.

*Use.* Modern writers do not allow Betony any considerable share of efficacy, although Antonius Musa, the physician sician of Augustus Cesar, filled a whole volume with an enumeration of its virtues, and supposed it to be a cure for no less than forty-nine disorders: Scopoli indeed says, he experienced its *cephalic* and *corroborant* effects, but its sensible qualities shew it to be more inert than most of the other verticillatæ. This plant and euphrasia both enter into the composition of Rowley's British herb tobacc6 and snuff. *Woodville.* The roots in a small dose have an *emetic* quality; and the powder of the dried plant is a good *errhine,* and readily promotes sneezing. *Lightfoot.* This plant was formerly much used in medicine, but it is discarded from the modern practice; however, it is not destitute of virtues, for when fresh it intoxicates, and the dried leaves excite sneezing. It is often smoaked as tobacco. Sheep eat it; goats refuse it.—*Withering.*

13. Stachys. *S. sylvatica.*

*Ang.* Hedge Nettle. Wound Wort.

*Gen. Desc. Bloss.* upper lip vaulted; lower lip bent back at the sides, the larger middle segment notched. Stamens after shedding, bent to the sides.

*Spec. Desc. Leaves* heart-shaped, on leaf-st. *Floral-l.* spear-shaped, pointed. *Stem-leaves* and *Cal.* hairy. *Flowers* six in a whirl, deep purple with white spots. *Woods, hedges.* Bl. July, Aug.

*Use.* This plant dyes *yellow.* It has a fœtid smell, and toads are fond of living under its shade. Sheep and goats eat it; horses, cows, and swine refuse it.—*Withering.*

14. Stachys. *S. palustris.*

*Ang.* Clown's Wound Wort. Clown's All-heal.

*Gen. Desc. As above.*

*Spec. Desc. Stem* four cornered, rough. *Leaves* strap-spear-shaped, half embracing the stem, sitting, in opposite pairs, very soft, unequally serrated. *Cal.* purple, set with fine hairs, ending in small globules. *Flowers* six to ten in a whirl, reddish purple, mottled; tube white; mouth compressed. *Watery places.* Bl. Aug.

*Use.* This plant has a fœtid smell and a bitter taste: it is reckoned a good vulnerary. In times of scarcity, the roots, which are sweet, have been used as food, either boiled, or dried and made into bread: swine are very fond of them.—*Lightfoot.*

15. Ballota. *B. nigra. Marrubium nigrum fœtidum.*

*Ang.* Stinking Horehound. Henbit.

*Gen. Desc.* Cal. salver-shaped, five teeth, and ten scores. Bloss. upper lip concave, scolloped.

*Spec.*

*Spec. Desc. Leaves* heart-shaped, undivided, serrated. *Cal.* teeth tapering to a point. *Bloss.* tube containing honey, closed above by five hairy tufts: upper lip hairy, not very entire, purple, variegated with white lines. *Var.* bloss. white. *Rubbish.* Bl. July, Aug.

*Use.* This plant stands recommended in *hysterical* cases. The Swedes reckon it an almost universal remedy in the diseases of their cattle. Horses, cows, sheep, and goats refuse it. —*Withering.*

16. Marrubium. *M. vulgare. M. album.*

*Ang.* White Horehound.

*Gen. Desc.* Cal. salver-shaped, rigid, ten scored. Bloss. up. lip. cloven, strap-shaped, straight.

*Spec. Desc. Cal. teeth* bristle-shaped, hooked; cal. woolly. *Leaves* wrinkled, hoary, thick veins beneath. *Whole plant* downy. *Bloss.* compressed, bowed, white: up. lip spear-shaped: lower lip, mid. segm. slightly scolloped; lat. segm. spear-shaped, short. *Anthers* with a black substance in the middle. *Road sides, rubbish.* Bl. July, Sept.

*Use.* The leaves have a strong aromatic but disagreeable smell, which by keeping is dissipated, and a bitter, penetrating, diffusive taste, durable in the mouth. It was formerly much extolled for its efficacy in removing obstructions of the lungs and other viscera; and has been chiefly employed in *humoural asthmas, obstinate coughs,* and *pulmonary consumptions:* instances are also mentioned of its successful use in *scirrhous affections of the liver, jaundice, cachexies,* and *menstrual suppressions.* Its virtues, however, though it does possess some medicinal power, are not clearly ascertained; and it is now rarely prescribed. A drachm of the dry leaves in powder, or 2 or 3 oz. of the expressed juice, or an infusion of half a handful of the fresh leaves, have been directed for a dose. The last mode is usually practised by the common people, with whom it is still a favourite remedy in *coughs* and *asthmas.* Bergius describes is as *tonic, emmenagogue* and *diuretic.* Dr. Cullen denies its virtues as a pectoral, and says that in several cases, it was judged hurtful. Taken in considerable quantities, it is said to loosen the body.—*Woodville.* It has a strong musky smell, and a bitter taste: it is reputed *attenuant* and *resolvent:* an infusion of the leaves in water, sweetened with honey, is recommended in *asthmatic* and *phthisicky* complaints, and most of the diseases of the lungs.—*Lightfoot.* It is a principal ingredient in the Negro Cæsar's remedy for vegetable poisons. A young man, having having taken mercurial medicines, was thrown into a salivation which lasted more than a year, while every method tried to remove it, rather increased the complaint; till at length Linnæus prescribed an infusion of this plant, and the patient was restored in a short time. Horses, cows, sheep, and goats refuse it.—*Withering.*

17. Origanum. *O. vulgare.*—*O. sylvestre. O. anglicum.*

*Ang.* Wild, or field marjoram.

*Gen. Desc.* Flowers forming a four-sided, spike-like cone.

*Spec. Desc.* *Spikes* roundish, panicled, clustered. *Floral leaves* egg-shaped, longer than the calyx. *Leaves* egg-heart-shaped, opposite, slightly serrated, dotted, hairyish. *Stem* woolly, coloured. *Cal.* nearly equal, mouth closed with bristly hairs; without beset with short fine hairs, and minute white shining globules. *Bloss.* pale red, hairy; middle segment longer than the rest. *Thickets, hedges, in calcareous soil.* Bl. July.

*Use.* It has an agreeable aromatic smell, approaching to that of the garden marjoram, and a pungent taste much resembling thyme; to which it is more nearly allied in its medicinal qualities than any other of the verticillatæ, and therefore deemed to be *emmenagogue, tonic, stomachic, &c.* These effects, however, can only be attributed to the aromatic and stimulant powers which all the herbs of this natural order seem to possess in common. Distilled with water, it yields a moderate quantity of very acrid penetrating essential oil, which has been much used for easing the pain of a carious tooth, by dropping it on cotton, and inserting it in the cavity. The dried leaves used instead of tea, are exceedingly grateful. They are also employed in medicated baths and fomentations.—*Woodville.* The whole plant is a warm aromatic: the essential oil of it is so acrid, that it may be considered as a caustic, and is much used with that intention by farriers. A little cotton wool moistened with it, and put into the hollow of an aching tooth, frequently relieves the pain. The country people use the tops to dye *purple.* Goats and sheep eat it; horses are not fond of it; cows refuse it.—*Withering.*

The medicinal qualities of the garden marjoram, or sweet marjoram. (O. marjorana) whose native country is not known, agree with those of its congener above, but being more fragrant, the sweet m. is deemed more *cephalic,* and better adapted to those complaints known by the name of nervous; and it may therefore be employed with the the same intentions as lavender. In its recent state, it is said (*Cohausen in Comm. Nor. a* 1742, *p.* 151.) to have been successfully applied to *scirrhous* tumors of the breasts. *Woodville.*

18. Thymus. *T. serpyllum.—Serpyllum vulgare minus.*

*Ang.* Common thyme. Wild thyme. Mother of thyme.

*Gen. Desc.* Cal. two-lipped; mouth closed with soft hairs.

*Spec. Desc. Flowers* in heads. *Stems* creeping, woody, nearly cylindrical. *Leaves* flat, blunt, fringed at the base, oblong-egg-shaped, entire, hollow dots on both surfaces. *Cal.* coloured, teeth fringed, a circle of white hairs inside round the base of the segments, expanding after the blossom falls. *Fil.* inserted below upper lip shorter, below under lip longer than the tube. *Bloss.* purplish red. *Heaths, mountainous places.* Bl. July, Aug.

*Use.* This plant, in common with the garden thyme, has an aromatic smell and a warm pungent taste; but though its sensible qualities are the same, it has a milder and more grateful flavor; is less medicinal; yields an essential oil in smaller quantity, and less acrid; and in its spirituous extract, comes greatly short of the penetrating warmth and pungency of the other.—*Lewis.*—*Woodville.* It has a pleasant aromatic scent; and is esteemed a good *nervine.* An infusion of it by way of tea, is reputed an almost infallible cure for the *Incubus* or night-mare.—*Lightfoot.* This fragrant plant yields an essential oil, which is very heating. An infusion of the leaves, removes the head-ache occasioned by the debauch of the preceding evening. A general opinion prevails, that the flesh of sheep, which feed upon thyme, is much superior in flavor to common mutton; but Mr. Bowles, in his account of sheep walks in Spain, (*Gent. Mag.* 1764.) points this out to be a vulgar error; he says that sheep will carefully push aside the thyme, and all other aromatic plants, to get at the grass growing underneath; and that they *never touch it,* unless when walking a pace, and then they will catch at any thing. The attachment of bees to this and other aromatic plants is well known. Goats eat it; swine refuse it.—*Withering.*

*Note.* There are 9 varieties of this species, for which See *Bot. Arr. l. c.*

( To be continued. )

